# Cranioplasty Following Decompressive Craniectomy

**DOI:** 10.3389/fneur.2019.01357

**Published:** 2020-01-29

**Authors:** Corrado Iaccarino, Angelos G. Kolias, Louis-Georges Roumy, Kostas Fountas, Amos Olufemi Adeleye

**Affiliations:** ^1^Neurosurgery Unit, University Hospital of Parma, Parma, Italy; ^2^Emergency Neurosurgery Unit, Azienda USL-IRCCS di Reggio Emilia, Reggio Emilia, Italy; ^3^Division of Neurosurgery, Addenbrooke's Hospital, University of Cambridge, Cambridge, United Kingdom; ^4^NIHR Global Health Research Group on Neurotrauma, University of Cambridge, Cambridge, United Kingdom; ^5^Department of Neurosurgery, Humanitas University and Research Hospital, Milan, Italy; ^6^Department of Neurosurgery, University Hospital of Larissa, University of Thessaly, Larissa, Greece; ^7^Division of Neurological Surgery, Department of Surgery, College of Medicine, University College Hospital, University of Ibadan, Ibadan, Nigeria

**Keywords:** cranioplasty, decompressive craniectomy, traumatic brain injury, cranial reconstruction, bone flap, posttraumatic hydrocephalus

## Abstract

Cranioplasty (CP) after decompressive craniectomy (DC) for trauma is a neurosurgical procedure that aims to restore esthesis, improve cerebrospinal fluid (CSF) dynamics, and provide cerebral protection. In turn, this can facilitate neurological rehabilitation and potentially enhance neurological recovery. However, CP can be associated with significant morbidity. Multiple aspects of CP must be considered to optimize its outcomes. Those aspects range from the intricacies of the surgical dissection/reconstruction during the procedure of CP, the types of materials used for the reconstruction, as well as the timing of the CP in relation to the DC. This article is a narrative mini-review that discusses the current evidence base and suggests that no consensus has been reached about several issues, such as an agreement on the best material for use in CP, the appropriate timing of CP after DC, and the optimal management of hydrocephalus in patients who need cranial reconstruction. Moreover, the protocol-driven standards of care for traumatic brain injury (TBI) patients in high-resource settings are virtually out of reach for low-income countries, including those pertaining to CP. Thus, there is a need to design appropriate prospective studies to provide context-specific solid recommendations regarding this topic.

## Introduction

The operative surgery of therapeutic decompressive craniectomy (DC) for traumatic brain injury (TBI) involves the elevation of a free cranial convexital bone flap that is stored either *in vivo* (e.g., abdominal or thigh subcutaneous pouches) or in *ex vivo* mediums (deep freezing and tissue banking) (1, 2). Skull defects can result from direct trauma or postsurgical craniectomy. Varying shapes, sizes, and complexities of the defect can be observed.

Cranioplasty (CP) after DC aims to restore esthesis (3), improve cerebrospinal fluid (CSF) dynamics, and provide cerebral protection. In turn, this can facilitate neurological rehabilitation and potentially enhance neurological recovery (3). Although regarded as a routine neurosurgical procedure, CP can be associated with significant morbidity (4, 5).

This paper is a narrative mini-review rather than a systematic review. Therefore, a full search strategy is not provided; rather, we mainly focused on articles in the English language published in PubMed during the last 15 years.

## Surgical Techniques

Although surgically straightforward, CP can lead to intraoperative complications at every operative step (elevation of the scalp flap, dissection of soft tissue from the underlying dura, and filling of the defect with a congruent rigid structure) (6, 7).

Required for flap elevation, the vascular territory of the flap must be kept in mind while performing the incision. Following an inner ellipse of the previous DC-surgery scar could contribute in most cases to the preservation of the vascular perfusion even if an incision outside of the ellipse might be needed in certain settings such as sinking skin flap syndrome (SSFS).

In patients where the skin may not be enough to cover the CP, due to an SSFS or skin lesions or scars, a single- or staged skin expansion procedure should be indicated.

However, most commonly, the operative dissections for CP after DC is performed by just opening the skin incision for the previous DC to develop the scalp flap for the CP.

The primary cranial damage control surgery is executed mainly by means of a unilateral frontotemporoparietal DC. This calls for a thoughtful consideration of the temporalis muscle (8), which is often found shrunken or inferiorly retracted toward its origins and adherent to the overlying scalp flaps and/or the underlying dura. This entails a delicate separation that can result in significant bleeding with a resulting increase in operative time. Other complications are intraoperative dural tears, cortical vascular, and parenchymal injuries, postoperative CSF wound leakage, as well as surgical site infections (SSI).

A number of techniques during the DC can potentially reduce the risks associated with the step of dissection. Some are preemptive techniques, such as the interposition of non-absorbable materials between the dura and the scalp flap during the primary DC and tagging the temporalis muscle with brightly colored, non-absorbable sutures for improved identification (9, 10).

## Complications

Routine CP is known to have a higher rate of postoperative complications than other elective cranial procedures (11), which may appear at any point during the clinical course due to various factors both directly and indirectly related to the CP itself.

Walcott et al. reported that previous reoperation, comorbid disease type, presence of a ventriculoperitoneal (VP) shunt, and general cardiovascular risk factors are predictors of complications of CP post-DC after stroke and trauma (12). Additionally, skin flap complications such as dehiscence, ulcers, and necrosis are reported (13) and may be related to the exposure of subjacent tissues, always occur after CP in unilateral craniectomy, and preferentially affect the temporoparietal region. However, no correlation has been found between the biomaterials used and skin complications. Dehiscences occur essentially due to poor preoperative conditions, such as in chronically sunken flaps. Ulcers were always associated with an underlying infection and were rarely observed in craniectomized patients before undergoing CP. Necrosis was ascribed to inadvertent sacrifice of the residual arterial supply after flap reopening or to a venous congestion.

### Infection

De Bonis et al. showed a 2.5-fold increased infection risk with a bifrontal CP compared with hemispheric/bihemispheric CP (14), regardless of the bone flap substitution material used. This is due to a longer incision and operative time, less temporalis muscle soft tissue coverage, and possible breaching of frontal sinuses during DC. Polymethylmethacrylate (PMMA) as the CP biomaterial shows significant infection rates when in contact with the nasal sinus mucosa or contaminated material (15). Hydroxyapatite (HA) implants (16) showed the highest incidence of infection (3.8%) in bifrontal defects. Also, a study of patients with titanium CP concluded that bifrontal insertion was one of the most relevant risk factors, with a complication rate of 40% including infections (17).

Following CP infection, the decision to remove a biomaterial is a complex issue and should be addressed in concert with plastic surgeons, especially when poor preoperative conditions of skin flap are observed. Even patients in good clinical conditions are at risk of sudden and/or further deterioration. Although, CP infection is rarely associated with sepsis, it is usually addressed by bone or implant removal until complete healing of the surgical field is achieved.

One possible way to address these issues of infection following CP is the development of new prosthetic biomaterials capable of resisting microbial colonization.

### Hydrocephalus (HC)

After DC, the occurrence of ventriculomegaly (VM) or HC is reported with varying incidences (10–45%) mainly due to differences in diagnostic criteria (18–21). The management of HC in patients in need of cranial reconstruction can be challenging and thus is not precisely defined. The debate mainly revolves around the timing of CSF diversion with respect to the CP.

Nasi et al. (22) reported 28.4% occurrence of HC in a series of 130 DC at 6.43 postoperative months. In 91.9% of patients, a ventriculoperitoneal shunt (VPS) was required, 76.4% of which was implanted after CP, 14.7% synchronous with, and only in 8.8% before the cranial reconstruction.

The disappearance of VM after CP is well-documented (23–25), and the postoperative management strategy of an unnecessary VPS placement (26–28) is yet unclear. In patients with a bulging scalp flap and VM, external CSF drainage achieved *via* ventriculostomy or lumbar drainage could allow an accurate repositioning of CP without brain damage.

The use of programmable shunts for patients dependent on CSF shunt has been effectively proven in various case series (29–31). Nevertheless, in socioeconomic environments with limited resources, a fixed pressure valve remains often the only option.

## Material Types

### Autologous Bone

With few exceptions, autologous bone remains the most commonly used material to fill cranial defects following DC (7, 32–34). It is biocompatible and quite cost-free. Whenever available, autologous bone thus remains the favored option for filling small- to medium-size defects, as well as even the large cranial defects following DC. It is however more likely to be associated with bone flap resorption (BFR) in the latter. The BFR is a non-linear process, which can result in structural breakdown of the CP requiring reoperation and even bone flap replacement with heterologous materials.

Korhonen et al. reported BFR as a complication occurring at various degrees in up to 90% of patients undergoing autologous CP after DC, in particular in patients younger than 30 years. In any case, it has been observed that postoperative monitoring for BFR required regular clinical follow-up, assessing for mechanical stability rather than routine CT.

Independent risk factors for reoperation were shown to be younger age, shunt dependency, and bone flap fragmentation due to a fracture. Hence, an initial artificial bone substitute implant rather than an autograft could be recommended in all patients with a fragmented flap (35).

Compared with synthetic biomaterials, the use of autologous bone for CP is associated with significantly increased odds of reoperation (36). However, autologous bone does not seem to increase infection rates compared with synthetic material. BFR is the main cause for reoperation overall (35).

Some authors raised the possibility that higher rates of complication in autologous bone graft would be partly explained by bone flap conservation methods. However, a systematic review performed by Corliss et al. found no such statistical evidence (37).

### PMMA

PMMA is a very common material for CP, and it can be found used as PMMA liquid or as solid PMMA customized implants. Intraoperatively, liquid PMMA takes from 10 to 20 min to be turned into a moldable viscous paste, which is then applied to the cranial defect (38). This process is an exothermic reaction from which the brain and the meninges need to be shielded. Liquid PMMA is non-absorbable, radiolucent, and inert. Additionally, it can be soaked with antibiotics, making it a good option for patients having failed multiple previous attempts at CP (38) because of SSI. It is both an effective and affordable choice for CP. The abovementioned exothermic reaction, intraoperative preparation, the relative contraindication in pregnancy, toxicity of fumes, as well as the need for artistic skills from the operators are all disadvantages.

On the other hand, solid custom-made PMMA, despite its cost, has a long-standing record, does not require to be prepared intraoperatively, does not cause any exothermic reactions, is easy to contour, is delivered sterile, as well as has a textured surface. To reduce the costs, the use of three-dimensional (3-D) patient-specific customized silicon molds is reported to be filled with less expensive liquid PMMA (39–41).

### Polyetheretherketone (PEEK)

PEEK has the advantage of being inert, pliable, and mechanically sound. It requires in-house sterilization and may increase seroma formation.

Punchak et al. (42) showed a trend toward decreased postoperative complication rates of PEEK CPs compared to autologous grafts and showed a stronger trend toward lower failure rates of PEEK grafts compared to titanium grafts. The overall complication rate was shown to be lower with PEEK than with titanium group (43).

### Titanium

Titanium can be manufactured as a plate, mesh, or 3-D porous implant and is available with varying stiffness and degrees of openness. Titanium is robust to resist secondary trauma while providing maximal stability of the cranial vault (44).

Titanium CP after DC is associated with better cosmetic and functional outcomes than primary autologous CP without increasing overall healthcare costs (20). Free flap coverage and soft tissue atrophy result in greater risk of titanium mesh exposure (45). The titanium mesh should be well-anchored onto the basi-temporal skull to avoid spontaneous fracture (46).

Most recently, 3-D porous titanium was implemented as a viable alternative. Despite its high cost and limited literature available, 3-D porous titanium shows promising results after a 1-year follow-up (47).

### Porous Hydroxyapatite (HA)

Porous HA shows biocompatibility due to its biomimetism and the absence of host immune interactions (48–50) or systemic/local toxicity (51). Composite biomaterials such as scaffolds surface-enriched HA nanoparticle using a poly(trimethylene carbonate) (PTMC) scaffold are shown to have a positive impact on bone generation and repair (45). Bony regeneration rates were reported in two patients having undergone CP at 6 months and 2.5 years, respectively (52). It is an appropriate material for use in large and complex cranial defect reconstruction (53).

A posttraumatic fracture rate of HA prosthesis is reported but, at the same time, HA has the ability to undergo self-repair (16, 53).

A study has tried to address the retention management of infection associated with hydroxyapatite CP (52). The suggestion is that a lower biofilm formation, lower rate of colonization compared to titanium (53), targeted antimicrobial therapy, and a satisfactory area of revascularization allow optimal antibiotic delivery on-site and were all decisive in the possibility of avoiding prosthesis removal.

### 3-D Prosthesis

Shape is another important factor for a successful CP as an increased congruence between the patient and the implant will lead to a better outcome overall as well as improved aesthetic benefit.

In neurosurgery, 3-D printing can be used to create prosthesis and molds used to reconstruct cranial defects using CT data to obtain the dimension and shape of the repair (54, 55). The cost of equipment, lack of knowledge and training, and introduction of commercial, FDA-approved media for printing are thought to be obstacles to a widespread adoption of neurosurgical 3-D printing usage (55).

## CP Following DC in Settings with Limited Resources

Until recently, the low-cost nature of the practice of neurosurgery in resource-limited regions meant that the costly protocol-driven standards of care for TBI in high-resource settings were virtually out of reach for most lower- to middle-income countries (LMICs).

However, it is now being increasingly recognized that when both clinical and radiological signs of a patient are in keeping with raised intracranial pressure (ICP) in TBI, the surgical procedure of DC should no longer be considered a last-tier treatment option. It can, and perhaps should, be performed sooner than later and most pragmatically so in these same low-resource LMICs where the other high-cost means of the non-surgical management of posttraumatic raised ICP are not available (56–59). There is therefore an increasing body of work on the use of DC in damage control surgery TBI from the developing countries.

**Figure 1:**
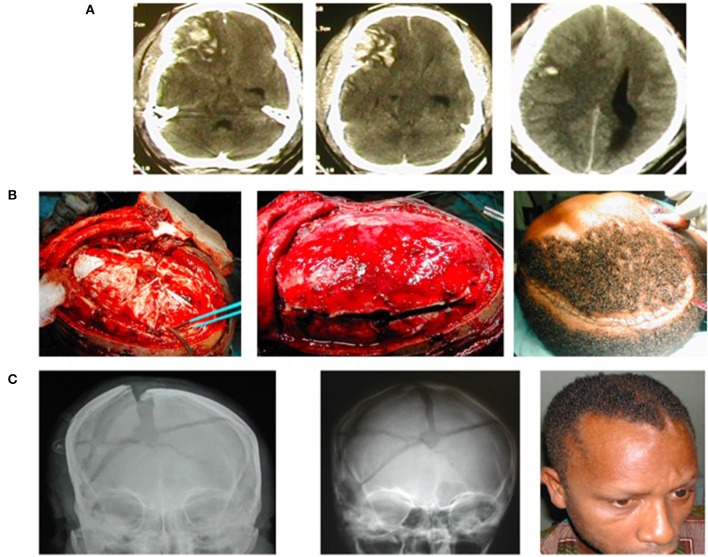
A young man suffered severe traumatic brain injury (TBI), GCS7/15, from road trauma. **(A)** Cranial CT showed right-sided brain contusions, acute subdural hematoma, subarachnoid hemorrhage, intracerebral hematoma, and bilateral brain swelling that was worse to the right—CT Rotterdam of 6. **(B)** Intraoperative image of the hinge craniotomy, cruciate durotomy, and evacuation of the extra-axial bleed (to the left); the bone flap returned, floating *in situ* (middle); and skin closure (to the right). **(C)** Plain skull X-ray on the first postoperative day showing the elevated/floating bone flap (to the left), bone also revealed comminuted skull fracture; Plain skull X-ray (middle) and clinical picture of the patient (right) 5 weeks postop showing the bone flap spontaneously returned to the rest of the cranium following resolution of the traumatic brain swelling.

Additionally, the surgical technique of *in situ* hinge DC (60–63) instead of the traditional DC has greatly influenced the literature of DC from the LMICs. Hinge DCs, also known as hinge craniotomies, by their nature do not as a rule require salvage CP. This would naturally be expected to be an attractive option as the surgical decompression of choice for raised ICP in the LMICs. There is thus a growing literature on the use of the hinge DC, including modifications of the originally described techniques (64–66) from these regions (Figure 1).

CP following DC as yet does not appear to be a major need in the LMICs. But whenever there is need to resort to the traditional DC (e.g., a forbidding massive brain swelling), then the autologous bone flap remains the overwhelming choice, and due to costs, PMMA is the second alternative.

## Timing of CP

The appropriate timing of CP after DC in relation to complication rate and outcome has yet to be established. The concept itself of early CP remains ill-defined and refers to time intervals varying from as little as 4 weeks up to as long as 12 weeks (Table 1).

**Table 1:** Studies evaluating timing of cranioplasty after craniectomy.

**References**	**Study design**	**No. of Pts**	**Age (mean)**	**Definition of early cranioplasty**	**Complication rate (%)**	**Notes**
Chun and Yi (67)	Restrospective cohort	45	49	<1 month	46.7	Early cranioplasty with significantly lower rate of complications (6.67% early, 53.3% late).
Chang et al. (68)	Restrospective cohort	212	44	<3 months	12.7	Early cranioplasty with significantly lower rate of complications (OR = 0.28, 95% CI 0.11–0.68).
Matsuno et al. (69)	Case-control	206	Range−6 months−79	NR	12.1	The mean time intervals after removal of bone flap of the infected group were longer than that of the non-infected group.
Waziri et al. (70)	Case-control	17	48	NR	47	Trend toward higher rates of post-cranioplasty hydrocephalus and longer time to cranioplasty.
Archavlis et al. (71)	Restrospective cohort	200	53	<7 weeks	9.5	Early cranioplasty may have better outcome (when no edema nor infection) but appear to increase risk of deep wound infections and osteomyelitis.
Schoekler et al. (72)	Restrospective cohort	58	46	NR	26.4	Tendency of resorption if cranioplasty performed more than 2 months after. No differences in the outcome.
Tasiou et al. (73)	Pubmed research				NR	Early cranioplasty may improve the outcome in selected cases.
Qasmi et al. (74)	Prospective cohort	30	32	<12 weeks	30	Early autologous cranioplasty offer acceptable neurological outcome.
Morton et al. (34)	Restrospective cohort	754	44	<1 month	24.6	Cranioplasty 15–30 days reduce infection, seizure, resorption, <90 days reduces hydrocephalus.
Beauchamp et al. (75)	Case-control	69	30	NR	39.1	No statistical significant difference in time to cranioplasty between those with and those without complications.
De Bonis et al. (14)	Restrospective cohort	185	All adults	<3 months	19.7	No significant difference in complication rates for early or late cranioplasty.
Gooch et al. (76)	Restrospective cohort	62	32	<1 month	33.8	OR for complications requiring reoperation was highest for patients undergoing cranioplasty 100–136 days after craniectomy.
Song et al. (77)	Restrospective cohort	43	NR	<12 weeks	NR	No effect on complication rate and global outcome by GOS.
Huang et al. (78)	Restrospective cohort	105	41.9	NR	9.5	Timing of cranioplasty is not related to outcome.
Piedra et al. (79)	Restrospective cohort (Vascular)	74	47	<10 weeks	18.9	Complication are similar for early and delayed cranioplasty.
Piedra et al. (80)	Restrospective cohort (Traumatic)	157	29.5	<12 weeks	35	Early cranioplasty does not alter the incidence of Complication.
Mukherjee et al. (17)	Retrospective cohort	144	41	<16 weeks	26.4	No difference in pre- and post-op GOS between time intervals.
Sundseth et al. (81)	Retrospective cohort (non-traumatic)	47	47.8	NR	26.4	Timing of cranioplasty is not related to the risk of infection
Kim et al. (82)	Retrospective cohort	85	50.3	<1 month	7.05	No statistical difference in infection rate between the 2 groups
Coulter et al. (83)	Restrospective cohort	166	39	NR	40.4	Timing of cranioplasty did not appear to be predictive of outcome.
Tsang et al. (28)	Restrospective cohort	NR	46.3	<3 months	16.7	Timing of cranioplasty had no significant association with complications.
Krause-Titz et al. (84)	Restrospective cohort	248		NR	18.5	Timing of cranioplasty had no significant influence on complications.
Schuss et al. (85)	Restrospective cohort	280	46	<2 months	16.4	Early cranioplasty with significantly higher rates of complications (25.9% early vs. 14.2% late).
Thavarajah et al. (86)	Restrospective cohort	82	NR	NR	11	Cranioplasty between 0 and 6 months had the greatest rate of infection.

*Early cranioplasty is not uniform among the various studies. Adapted from Piedra et al. (79)*.

Timing varies according to three pre-CP scenarios encountered, setting the earliest time at which a CP can be performed.

**Type 1:** The brain is depressed with a significant sinking of the post-DC flap due to a posttraumatic brain atrophy or overdrainage of a ventriculoperitoneal shunt (VPS). Thus, a pre-CP long-lasting CSF diversion should be avoided.

**Type 2:** The post-craniectomy scalp flap is at the same level as the margins of the cranial vault. The brain should be in the most physiological condition thus at a low risk of observing the development of HC and/or postoperative blood collections.

**Type 3:** The post-craniectomy scalp flap is over the cranial vault margin due to brain swelling and/or HC/VM. This could well be the worst scenario due to the difficulty of diagnosis and treatment.

Infection was reported to be the highest risk within 14 days of craniectomy, HC within 90 days, and seizure risk after 90 days. Hence, some advocate for an ultra-early CP taking place between 15 and 30 days that would minimize infection, seizure, and autologous flap resorption risks.

In a retrospective cohort study (71), the functional outcome was found to be better for CP performed at the <7 weeks and at 7–12 weeks group compared with the >12 weeks group. Nevertheless, the authors stressed an earlier time to CP should be set as soon as brain edema had normalized so as to have higher chances of a better neurologic outcome and not apparently increased infection rate. At the same time, CP performed at <7 weeks was associated with a significant increase in infection rates when comorbidities, such as diabetes, thromboembolism, and colonization with multidrug resistant (MDR) pathogens, were present (87). Thus, both clinical status and infective status are strong determinants of the outcome of an early CP (<7 weeks) and are of paramount importance in establishing the timing of an early CP.

**Figure 2:**
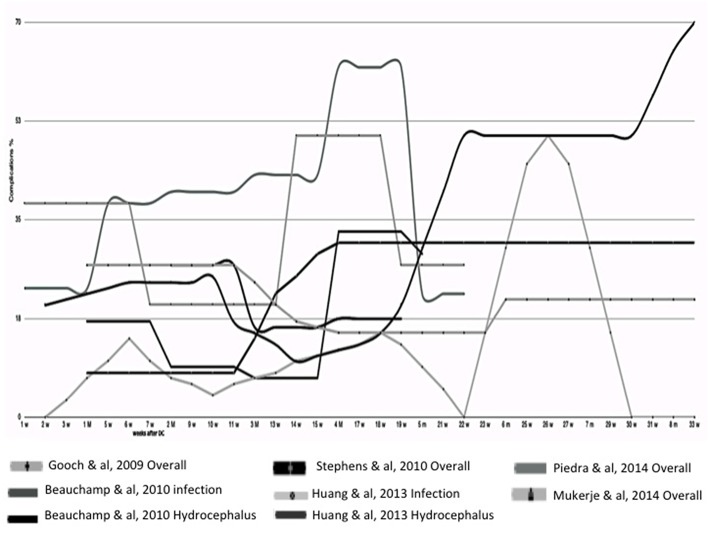
Timing of Cranioplasty. In this graph are compared different clinical courses after cranioplasty analyzed from different papers, where the timing of onset of complication is well-reported. In this partial analysis of literature data, a higher rate of complication is suggested when cranioplasty has been performed between the third and fifth month.

Conclusions regarding early CP vary widely among different studies (75, 76, 88). Some authors attribute a lower rate of complications to an early CP (67–69, 71, 73, 74), others describe a lower risk of hydrocephalus (34, 70), while no improvement in outcome following early CP was also found (72, 78). Moreover, no impact of timing on outcome (17, 83) or complications (3, 14, 28, 77, 79–82, 84) have been reported. Only two authors (85, 86) associated an early CP with poor outcome, in particular, the highest rate of infection between 0 and 6 months (86). An analysis of literature data suggests a higher rate of complication when CP has been performed between the third and fifth month (Figure 2).

Last reported systematic review and meta-analysis (3) suggested that early CP may lead to even greater improvements. Nevertheless, despite a growing consensus that earlier is better, no more than low-level evidence from retrospective, poorly matched cohort studies (Class IIb, Level C) has been published on this subject.

## Conclusion

Despite its therapeutic and cosmetic advantages, CP following DC is not reported to correlate strongly with improved neurologic rehabilitation and outcome.

Different surgical approaches can be used to reduce the surgical complications that may arise at any point of the clinical course among which bifrontal CP is a strong independent risk factor.

Autologous bone is the most commonly used material despite its association with BFR and higher rate of implant failure requiring removal.

A consensus has yet to be reached with regard to the best heterologous material for CP. Porous prostheses may offer promising results despite higher costs.

Standards of care for DC are not applicable in LMICs due to high costs, and thus autologous bone grafts are favored.

Regarding HC, the optimal timing for shunting is yet to be firmly defined. A one-step surgery with CP and CSF shunting and a two-step surgery with or without external CSF drainage are reported as alternatives of management. Finally, CSF shunting without a timely CP should be avoided.

While waiting for results of an ongoing randomized controlled trial (RCT) on early vs. late CP promoted by NIHR Global Health Research Group on Neurotrauma, the timing of reconstructive CP should rather be based on an objective case-by-case assessment of the neurological status of each patient, resolution of brain swelling, and complications associated with large calvarial defects rather than arbitrary time windows (73) and should be performed as soon as brain swelling resolves on CT scan, provided that the patient is not in an infectious state (89).

The authors are aware that the results of future studies may dictate updating many of the recommendations on several aspects of CP after DC contained in this review.

## Author Contributions

CI, AK, AA, and L-GR: writing manuscript. CI and AA: analysis of literature. CI and AA: collecting cases. AK and KF: supervision of manuscript.

### Conflict of Interest

CI is a consulent for post market surveillance for Finceramica S.p.A. The remaining authors declare that the research was conducted in the absence of any commercial or financial relationships that could be construed as a potential conflict of interest.
